# UV-Stressed *Daphnia pulex* Increase Fitness through Uptake of Vitamin D_3_

**DOI:** 10.1371/journal.pone.0131847

**Published:** 2015-07-06

**Authors:** Sandra J. Connelly, Kelly Walling, Steven A. Wilbert, Diane M. Catlin, Cailin E. Monaghan, Sofiya Hlynchuk, Pamela G. Meehl, Lauren N. Resch, J. Valerie Carrera, Stephanie M. Bowles, Michael D. Clark, Loraine T. Tan, Jeremy A. Cody

**Affiliations:** 1 Thomas H. Gosnell School of Life Sciences, Rochester Institute of Technology, Rochester, New York, United States of America; 2 School of Health Science and Technology, Rochester Institute of Technology, Rochester, New York, United States of America; 3 College of Imaging Arts and Sciences, Rochester Institute of Technology, Rochester, New York, United States of America; 4 School of Chemistry and Materials Science, Rochester Institute of Technology, Rochester, New York, United States of America; Federal University of Rio de Janeiro, BRAZIL

## Abstract

Ultraviolet radiation is known to be highly variable in aquatic ecosystems. It has been suggested that UV-exposed organisms may demonstrate enough phenotypic plasticity to maintain the relative fitness of natural populations. Our long-term objective is to determine the potential photoprotective effect of vitamin D_3_ on *Daphnia pulex* exposed to acute or chronic UV radiation. Herein we report our initial findings in this endeavor. *D*. *pulex* survival and reproduction (fitness) was monitored for 5 d as a proof of concept study. Significantly higher fitness was observed in the *D*. *pulex* with D_3_ than those without (most extreme effects observed were 0% survival in the absence of D_3_ and 100% with 10 ppm D_3_). Vitamin D_3_ was isolated from the culture media, the algal food (*Pseudokirchneriella*), and the *D*. *pulex* and quantified using high performance liquid chromatography (HPLC). Vitamin D_3_ was fluorescently labeled using a phenothiazinium dye and added to cultures of *D*. *pulex*. Images demonstrating the uptake of D_3_ into the tissues and carapace of the *D*. *pulex* were acquired. Our initial findings suggest a positive role for D_3_ in ecosystems as both UV-stressed algae and *Daphnia* sequester D_3_, and *D*. *pulex* demonstrate increased fitness in the presence of D_3_.

## Introduction

Decreased stratospheric ozone has historically altered the ultraviolet radiation (UVR) penetration of the atmosphere [1] and current elevated UVR conditions are likely to persist at least through mid century [2]. DNA is thought to be the primary target of UVR damage and induction of damage in DNA is linearly related to UVR exposure (dose) in isolated DNA [3], but has been more recently associated with overall physiological effects in multiple organisms, particularly in high UVR regions (e.g. [4]). Less is known about the extent of variability in net DNA damage in natural systems, particularly those under variable inputs from the surrounding terrestrial environments.

UVR is known to play a critical role in the formation of cancer in humans, especially skin cancer [5–7], and UVR can directly damage the DNA of aquatic organisms by the same mechanism. This damage in aquatic organisms presumably results in protective physiology and behavior that minimizes genomic damage and death [8–12]. Protection from UVR may be accomplished by several primary means: behavioral avoidance of detected UVR, increased levels of photoprotection through UVR-absorbing molecules (e.g. melanin), repair of the damaged DNA, or increasing tolerance to the UVR exposure. All of these options, however, are physiologically costly to the organism [3], at least at some level.

The basic mechanisms of UVR protection, avoidance, photoprotection, and repair, can be observed in aquatic organisms. Behavioral avoidance and increased levels of photoprotective compounds are some of the more thoroughly studied mechanisms. Some freshwater zooplankton, such as *Daphnia* spp., are well-known for altering their vertical migration patterns to avoid intense UVR exposure [13] due at least in part to detection of UVR (e.g. negative phototaxis is wavelength dependent in *Daphnia* [14]; observed morphological changes in crustacean retinas [15]). Rhode et al. [16] determined a direct relationship between the exposure of *Daphnia* sp. to UVR and their depth of vertical migration. In addition, the authors reported an inverse relationship between this response to UVR exposure and the level of pigmentation that the species possess. Increased melanin concentrations in *Daphnia* sp. with increased UVR exposure is seen repeatedly in the literature (e.g., [17, 18]). These two means of protection are physiologically costly and make the organism more susceptible to predation by altered migration patterns and increased coloration, concomitantly increasing the visibility to predators and affecting the survival rate of the zooplankton species [19]. In the end, an individual’s ability to avoid and repair DNA damage will ultimately determine the severity of any exposure, and have a direct effect on the survival and reproduction of the organism.

**Fig 1:**
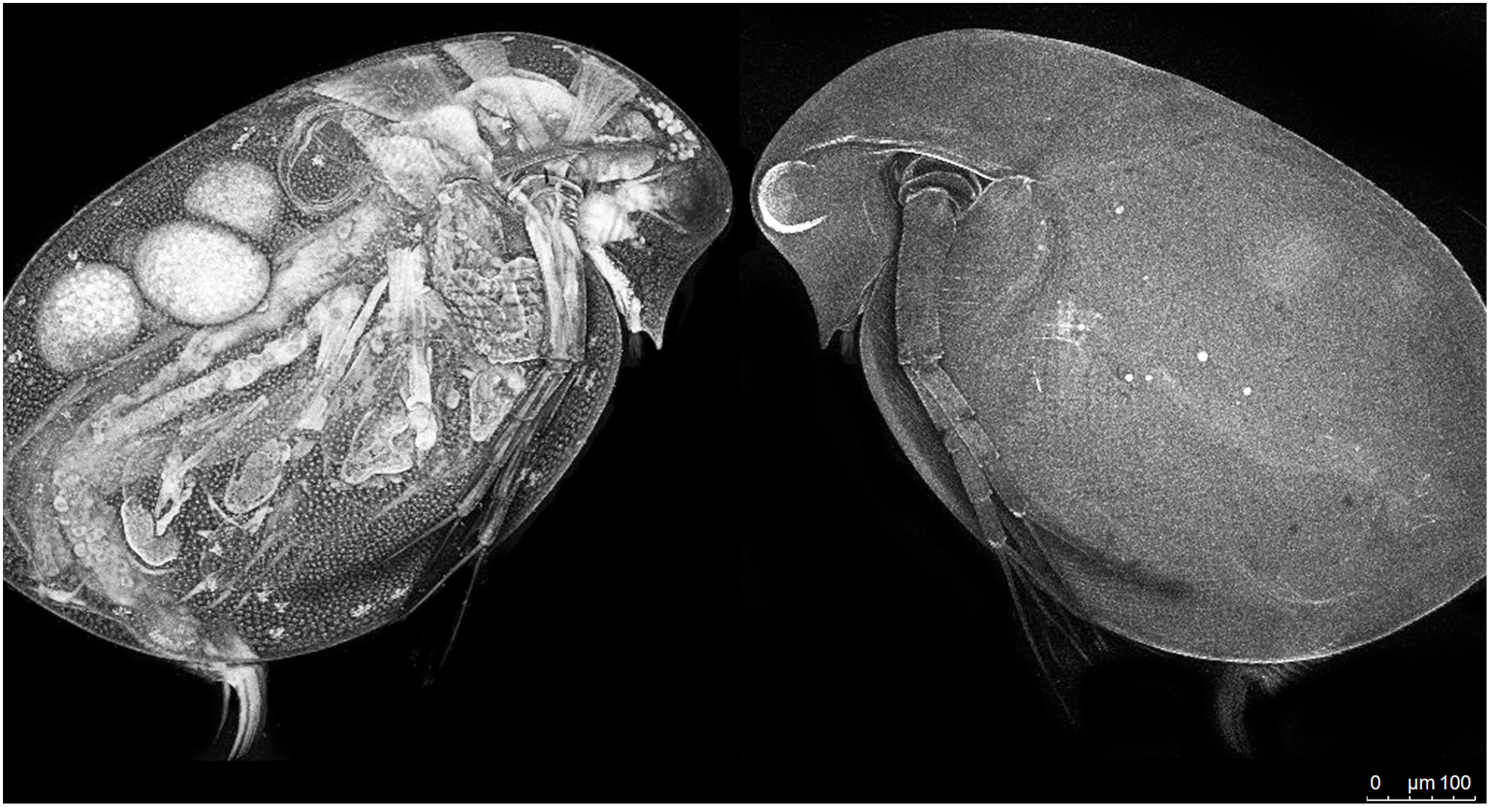
D. pulex. *D*. *pulex* were exposed to ethylene blue for 15 min, rinsed thoroughly, and imaged using a Maximum Intensity Projection Z Plane Stack (10x, Leica SP5 Scanning Laser Confocal Microscope). The intensity image presented here was used as proof of concept during the characterization of ethylene blue.

For aquatic invertebrates, such as *Daphnia* (Fig 1), with potentially high UVR exposure rates and short lifespans, minimizing the accumulation of damaged cells (DNA) is critical. The overall survival and reproduction rates (fitness) of an organism should increase if the accumulated damage is less physiologically costly than avoiding and/or repairing the damage (“net effect”). How can organisms mitigate this damage and repair without increasing the overall physiological costs to the point that their fitness is decreased? “Transparent” compounds such as mycosporine-like amino acids (MAA) [20] are believed to play a significant role in photoprotection of aquatic organisms as opposed to an increase in pigmentation, which is understood to increase predation rates [19]. Known photoprotective compounds are likely not the whole story for aquatic organisms. Previous studies have reported enhanced survival and reproduction of invertebrates with an increase in vitamins and/or antioxidants (e.g. B_12_ is required in *Daphnia pulex* for the development of viable offspring [21]; vitamin D is required to maintain fecundity in *Moina* [22]); antioxidants are required by *Chasmagnathus granulata* following UV-induced DNA damage [23]). It is theorized that vitamins taken up from the environment, either from natural production or terrestrial runoff, may play an important role in the overall fitness of an organism. If the vitamins are sequestered and used directly or indirectly to the benefit of the organism (decreased net damage, increased survival and reproduction), understanding the role of these compounds is crucial to assessing the ultimate impact of UVR on aquatic organisms and the overall ecosystem structure.

This study explores the possible mitigation of UVR-induced damage in *Daphnia pulex* in the presence of vitamin D_3_ (molecular structure; S1 Fig)–on the basis that D_3_ will contribute to the overall fitness of the organism through its uptake directly (in suspension) and/or indirectly (in the algal food source) from the aquatic environment. “Fitness” shall be used herein as a measurement of the survival and reproduction rates of the treated organisms versus the untreated controls within the time course of the experiment, and is not suggested as a point for which population fitness should be extrapolated beyond the limits of the experiment. While D_3_ has well-known benefits to vertebrates exposed to UVR, its role in UVR exposed aquatic invertebrates is not well documented. The photoprotective potential reported herein will be represented as the quantitative difference between the fitness of *D*. *pulex* in the presence and absence of D_3_ and UVR while other variables (e.g. nutrients and temperature) are held constant.

The direct effects of environmental vitamin D_3_ uptake on *D*. *pulex* exposed to UVR are examined using a three-tiered approach. First, from a big picture perspective, we consider the direct effect of D_3_ on *D*. *pulex* populations, and ask the question: what concentration, if any, of vitamin D_3_ will increase the fitness of *D*. *pulex* in the presence of acute or chronic UVR? Secondly, focusing on the individual organisms, we quantify vitamin D_3_ in the *D*. *pulex*, the *Pseudokirchneriella*, and the culture media of the *D*. *pulex* using high-performance liquid chromatography (HPLC), and ask the questions: is vitamin D_3_ sequestered from the environment by the *Daphnia* and / or the algae, and what effect might that have on the fitness of the *D*. *pulex*? Lastly, narrowing in on a single individual, using phenothiazinium (fluorescently) labeled vitamin D_3_ and confocal microscopy, we ask the questions: is vitamin D_3_ moved from the gut of the *D*. *pulex* into the surrounding tissues, and, if so, could this permit greater access of the *D*. *pulex* to the molecule and improve individual fitness?

## Materials and Methods

### *Daphnia* rearing conditions with vitamin D_3_

*D*. *pulex* were originally purchased from Aquatic BioSystems, Inc. (http://www.aquaticbiosystems.com/) in 2009 and have since been maintained in culture in the Connelly Molecular Ecology Lab at Rochester Institute of Technology. Subsample clone lines of the Connelly Lab stock cultures were isolated and cultured at 20°C for 6 generations prior to the UVR–fitness experiments. The cultures were maintained in 500 mL beakers on a 16:8 light:dark cycle with Caron Cool White standard light bulbs (0.11 kJ/m^2^/day UV-A with no detectable UV-B or UV-C; Spectroline DRC Multi-wavelength Radiometer with XTS 254 nm, 300 nm, and 365 nm sensors) in a Caron Model 6022–1 Diurnal Incubator. The cultures were grown in synthetic freshwater (US Environmental Protection Agency; approved culture media for *Daphnia* that mimics natural water with known micro and macronutrient composition; Moderately Hard Synthetic Water, pH = 7.4–7.8; http://water.epa.gov/scitech/methods/), fed 1.5e7 cells *Pseudokirchneriella subcapitata* (green microalgae formerly known as *Selenastrum*) every 48 h, and population counts were maintained below 25 *Daphnia* per 100 mL to ensure healthy individuals for experimentation. When UVR experiments were set up, 5 d old juveniles were selected from cultured populations, and randomly distributed into the exposure vessels (across treatments) to eliminate experimental selection bias of the *Daphnia*. While UVR tolerance of *Daphnia* spp. is recognized to vary with age (e.g., [8]), the experiments herein focus on the age bracket (pre-reproductive) that will most significantly influence the population in the short and long term.

### Chronic (UV-A) exposures

As D_3_ is extremely hydrophobic, the maximum concentration of D_3_ (Cholecalciferol, >98%, cat. no. C9756, Sigma-Aldrich, USA) that was feasible in the experimental vessels (e.g. did not coat the surface water) was 10 mg/100 mL. Concomitantly, 5 mg and 10 mg D_3_ / 100mL additions were chosen, as there was no visually observable D_3_ floating on the surface (observed by naked eye and 4x magnification of experimental vessels). The background control was 0 mg D_3_ to assess the natural D_3_ production by the algae in all reported experiments.

The UV-A source for this experiment was 2 Q-Lab UV-A 340 lamps (QUV-UV-A340, Q Lab, Westlake, OH, USA; http://www.q-lab.com/documents/public/d6f438b3-dd28-4126-b3fd-659958759358.pdf) yielding an overall spectral output of 295–365 nm. In addition, 2 CoolWhite bulbs (Philips, 32W) were also used to more closely mimic natural conditions (broad spectrum). For the chronic UV-A exposures, 6 culture vessels (266 mL plastic cup, Solo TP9), each containing 1 *D*. *pulex* juvenile (to monitor reproduction per individual *Daphnia*), 100 mL synthetic freshwater, 1 mL *Pseudokirchneriella* (1.5e7 cells), and vitamin D_3_ (0–10 mg) were placed beneath the UV-A + CoolWhite lamp assembly. Samples were exposed for 5 d, a standard exposure duration to minimize artificial spectral variation [24], yielding a dose of 115 kJ/m^2^/nm at 365 nm (Spectroline DRC Multi-wavelength Radiometer with XTS 365 nm sensor measurements made at the bench top–bottom of the culture vessel–where *Daphnia* were most commonly located). All exposures were conducted at the same temperature (20°C) and in the same culture conditions (water and algae) with 0, 2, 4, 6, 8, or 10 mg vitamin D_3_/100 mL additions being the only variable. The *Daphnia* were fed 1.5e7 cells *Pseudokirchneriella* every 24 h for the duration of the experiment and at the same time the fitness of the *D*. *pulex* was assessed, with survival and reproduction results tabulated. Three trials of this experiment were conducted, non-sequentially, and the results are presented as average percent survival of the *D*. *pulex* across experiments, and average reproduction per individual *D*. *pulex*.

### Acute (UV-A and UV-B) exposures

The acute UV-A source for this experiment was 1 Spectronics Spectroline XX-15A 15 W lamp housing with 2 bulbs peaking at 365 nm (Westbury, NY, USA). For these exposures, 6 quartz Petri dishes (Quartz Scientific, Fairport Harbor, OH, USA), each containing 1 *D*. *pulex* juvenile and 30 mL synthetic freshwater, were placed beneath the lamp housing. Samples were exposed for 0, 5, or 10 min, with or without screening (stainless wire mesh, 60x60 mesh per linear inch transmitting 37.5%; McMaster Carr cat. No. 9238T336, Princeton, NJ, USA) yielding 7 UV-A doses ranging from 0–54 kJ/m^2^/nm (Spectroline DRC Multi-wavelength Radiometer with XTS 365 nm sensor). All exposures were conducted at the same temperature (20°C) and in the same culture conditions (water and algae) with total UV-A exposure as the only variable (0 kJ/m^2^ treatment represents the no UVR exposure control). Following acute exposure, all *Daphnia* were transferred to individual culture vessels (1 *D*. *pulex* per vessel) containing 100 mL synthetic freshwater, 0–10 mg of vitamin D_3_ was added, and the *D*. *pulex* were fed 1.5e7 cells *Pseudokirchneriella*. It was decided to add vitamin D_3_ after the UVR exposure as the vitamin is known to be quite photoreactive, and the experiment was focusing specifically on tracking the vitamin D_3_, not any photoproducts at this time. The experimental vessels were then placed under CoolWhite lamps (Philips, 32W). The experimental conditions and data collection were conducted exactly as in the chronic UV-A exposures.

The only notable difference between the design of UV-A and UV-B acute exposure experiments was the UVR source itself with no other changes to the protocol. The acute UV-B source for this experiment was 1 Spectronics Spectroline XX-15B 15W lamp housing with 2 bulbs peaking at 312 nm (Westbury, NY, USA) yielding a spectral output of 281–405 nm. Samples were exposed for 0, 5, or 10 min yielding a UV-B dose of 0, 1.59, or 3.18 kJ/m^2^/nm, respectively (Spectroline DRC Multi-wavelength Radiometer with XTS 300 nm sensor).

### Quantifying vitamin D_3_ in *Daphnia* and algae using HPLC: *Daphnia* methods

High-Performance Liquid Chromatography is a common analytical method used to separate, identify and quantify compounds in a solution. This experimental design used HPLC to quantify vitamin D_3_ present in *D*. *pulex*, the microcosm (culture) water, and the algae (the potential intermediate consumer and/or producer of vitamin D_3_). 50 mL conical experimental tubes were established with 49 mL of synthetic freshwater, 20 *D*. *pulex*, 1 mL of *Pseudokirchneriella* (1.5e7 cells), and D_3_ as powder to the labeled tubes at 0, 5, *or* 10 mg, as appropriate. The tubes were loosely covered with Parafilm, placed in a rack on a rocker table parallel to a CoolWhite fluorescent bulb (Philips, 32W) and 2 Q-Lab UV-A 340 lamps (QUV-UV-A340, Q Lab, Westlake, OH, USA) yielding a spectral output of 295–365 nm on a 16:8 light:dark cycle at 20°C, and left for 72 h. At 24 h and 48 h 1 mL of *Pseudokirchneriella* (1.5e7 cells) was added to each tube to provide an additional food supply for the *Daphnia*.

After 72 h, each experimental replicate was divided into three samples: *Daphnia*, algae, and aqueous. The *D*. *pulex* were removed from the tubes by pipette (without aqueous media), frozen using liquid nitrogen, and ground using a mortar and pestle in preparation for D_3_ extraction. The algae were separated from the aqueous media by centrifuging all remaining contents of the 50 mL conical tubes at 2,500 g for 5 min. The aqueous samples were collected from these tubes as supernatant using a 50 mL borosilicate glass pipette, and the algae were rinsed from the bottoms of the centrifuge tubes using synthetic freshwater media during the extraction procedure. D_3_ was extracted from the *Daphnia* and algae samples using a triplicate ethyl acetate rinse. D_3_ in the aqueous samples was extracted with ethyl acetate (3 x 20 mL) and titrated. All samples for analysis were collected, filtered (Whatman filter paper, grade 1, 0.11 μm, 4.25 cm diameter), and then concentrated using a rotary evaporator. After concentration, residues of *Daphnia*, algae, and aqueous samples were diluted to a known final volume with 100% HPLC grade methanol (typically 10 mL for *Daphnia* and algae samples, and 20 mL for aqueous samples, to be within range of the standards). A vitamin D_3_ stock solution was prepared by diluting the appropriate mass of solid in 100% HPLC grade methanol. A ten-point calibration curve was prepared in addition to a methanol blank.

The method for HPLC determination of D_3_ was modified from Brunetto, et al. [25]. All samples were syringe filtered and an aliquot of each filtered sample was placed in an auto-sampler vial. Samples were stored at 4°C and protected from light until chromatographic analysis. All chromatography was completed within 2 d of sample preparation. Results are reported as the average (triplicate) for a series of experiments under a given set of conditions. All error bars represent ± 1 standard deviation.

The analysis of samples was carried out using an Agilent 1100 HPLC system equipped with UV-Vis. Separation was achieved in a reversed phase silica gel C18 column (150 x 4.6 mm length and internal diameter, 5μm pore size). Before each analysis the column was cleaned and regenerated using 100% HPLC grade methanol. The mobile phase of the HPLC system consisted of 1% acetic acid in HPLC grade water (solvent A) and HPLC grade methanol (solvent B). The isocratic mobile phase was set at 95% solvent B. The total flow rate of the mobile phase was 1 mL/min and the sample volume injected was 50 μL. The run duration was a total time of 12 min with a 2 min post time. The column temperature was controlled at 25°C. The detection wavelength was 287 nm, the absorbance maxima of vitamin D_3_ stock solution. All mobile phase solutions were vacuum filtered prior to experimentation. Purging of the lines and column was completed using HPLC grade water, followed by solvent B at the conclusion of each experiment sequence.

### Algae methods

To quantify vitamin D_3_ in algae, and determine the contribution of the algae to the production of D_3_ in the microcosms, *Pseudokirchneriella* was added to 50 mL conical centrifuge tubes in volumes 0, 2, 4, 6, 8, and 10 mL and diluted with synthetic freshwater to a final volume of 50 mL. D_3_ powder (0, 5, or 10 mg) was added to the appropriate tubes. The tubes were sealed with Parafilm, placed in a rack on a rocker table parallel to a Cool White fluorescent bulb (Philips, 32W) on a 16:8 light:dark cycle at 20°C, and left for 72 h.

Sample extraction and HPLC analysis was conducted in a similar manner as stated in the *Daphnia* methods section with only slight modifications. The mobile phase consisted of 0.1% acetic acid in HPLC grade water (solvent A) and 0.1% acetic acid in HPLC grade methanol (solvent B). The mobile phase gradient increased from 80–100% solvent B for the first 7 min then was held constant at 100% for 3 min, at which time the mobile phase was returned to starting conditions. The total flow rate of the mobile phase was 1.5 mL/min and the sample volume injected was 100 μl.

### Synthetic organic chemistry methods

**Fig 2:**
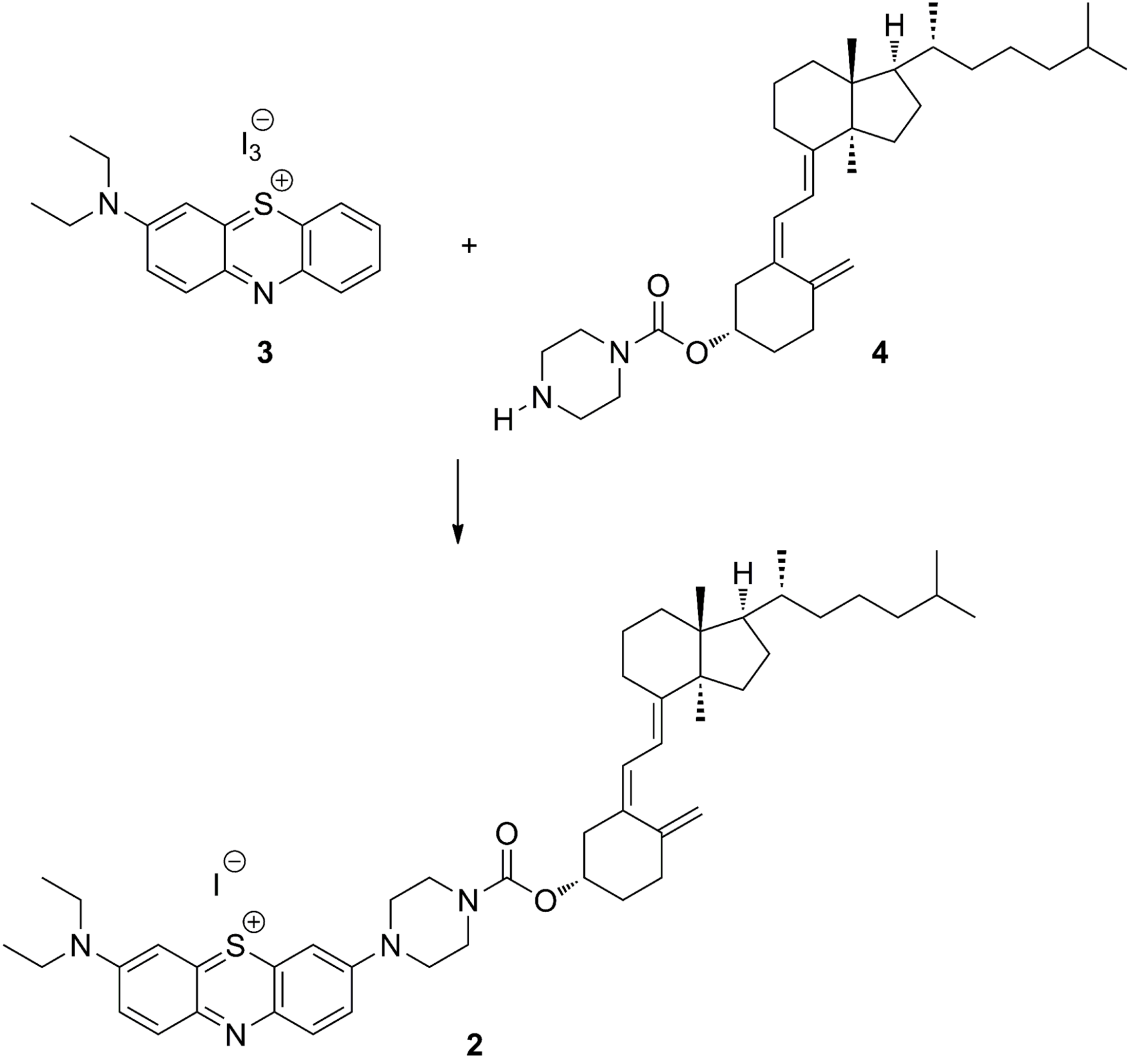
Synthetic route for the preparation of fluorescently labeled vitamin D_3_. The carbodiimidazole (CDI) coupling of vitamin D_3_ and the fluorescent dye linker.

To prepare fluorescently labeled vitamin D_3_ **2**, an appropriately functionalized fluorescent dye **3** [26] and vitamin D_3_ with a linker appendage **4** were synthesized and coupled (Fig 2). The functionalized fluorescent dye **3** (Fig 2) was prepared in two steps. The first step was initiated by adding iodine (16.2 g) as a solid portion-wise to a solution of phenothiazine (4.0 g) in dichloromethane (400 mL). The mixture was stirred for 12 h and then the phenothiazin-5-ium tetraiodide hydrate solids were filtered to provide a fine dark blue solid (16.33 g). The solids were then washed with dichloromethane (3 × 25 mL). A portion of the solids (9.6 g) was transformed further by stirring as a solution in methanol (88 mL) and diethyl amine (3.5 mL) at ambient temperature in the absence of light. After 7 h the precipitate was filtered and washed with methanol (3 × 43 mL) to give the functionalized fluorescent dye (Fig 2: **3**; 8.3 g) as a dark blue solid.

The linker was attached to the alcohol functionality of vitamin D_3_ by a carbodiimidazole (CDI) coupling. First vitamin D_3_ (4.0 g) was added to a solution of CDI (5.5 g) and 4-dimethylaminopyridine (DMAP, 0.07 g) in dichloromethane (42 mL). The reaction was heated to reflux under argon atmosphere. After stirring at reflux for 90 min, piperizine (2.25 g) was added and refluxed for an additional 12 h. The reaction was worked up by washing the mixture with water (2 × 14 mL) and a saturated brine solution (14 mL). It was then dried over magnesium sulfate, filtered, and concentrated to give 6.3 g of an oil. The crude oil was purified by silica gel flash column chromatography (ethyl acetate/hexanes) to provide **4** (Fig 2; 5.1 g, 98% yield) as a yellow oil.

Linking vitamin D_3_ **4** and fluorescent dye **3** was accomplished by stirring a solution of the two compounds together (Fig 2). Under argon atmosphere, piperizine functionalized vitamin D_3_ **4** (3.74 g) in dichloromethane (53 mL) was added drop wise over a course of 19 min to a solution of **3** (4.9 g) in dicholoromethane (53 mL) and triethyl amine (five equivalence, 5.26 mL). The reaction was then heated to reflux and stirred at reflux for 150 min. The reaction was allowed to cool to room temperature, concentrated down to 10 mL, and added slowly to 250 mL of diethyl ether using an addition funnel. The resulting precipitate was filtered and washed thoroughly with diethyl ether. The collected solids (3.7 g) were slurried in tetrahydrofuran (20 mL), filtered, and washed with an additional 20 mL of tetrahydrofuran. The filtrate was concentrated *in vacuo* to provide **2** (2.52 g, approximately 32% pure) as a blue powder. The purity was determined based on ^1^H NMR integration ratio of alkene protons and the major impurity, triethyl amine.HI salt.

The fluorescent properties and uptake of labeled vitamin D_3_ **2** were compared with ethylene blue (S2 Fig: **5**). Ethylene blue was prepared as described by Cody et al. [20]. This dye was selected as the fluorescence did not interfere, nor was it confused, with the autofluorescence of the *Daphnia* and the algae in the imaging experiments to be described.

Samples of labeled vitamin D_3_ (Fig 2: **2**) and ethylene blue were prepared by dissolving the appropriate amount of solid in ethanol. Samples were placed into quartz cuvettes and spectroscopic characterization was completed in two ways. UV-Vis absorbance spectra were recorded by using a Shimadzu UV-2401 PC spectrophotometer. A 50 W halogen lamp and D_2_ lamp were used as the light source. The spectral bandwidth was maintained at 1 nm, using a fast wavelength scanning speed and a sampling internal of 0.5 nm. Steady-state emission spectra were recorded using a Horiba Jobin Yvon FluoroMax-4P spectrofluorimeter. A 150 W Xenon arc lamp was used as the excitation source. The spectral bandpass was maintained at 2 nm for both excitation and emission monochromators. All samples were protected from light until spectroscopic analysis. All analysis was completed within 1 d of sample preparation.

### UV-Vis and fluorescence characterization

*Daphnia* are notoriously cumbersome to work with in fluorescent imaging techniques, due to the vast quantity of autofluorescent materials in their tissues and the algal food sources in their guts. By this, accurate characterization of fluorescent compounds **2** and **5** was critical for their use in tracking vitamin D_3_ in the *D*. *pulex*. The fluorescently labeled vitamin D_3_ (excitation = 650nm; S4 Fig) has a relatively narrowband fluorescence peak in the red region, making it easy to detect despite the presence of *Daphnia* autofluorescence in the blue region. Further, the linking of the ethylene blue to the D_3_ has minimal effect on its spectroscopic properties. This permitted working within the narrow fluorescence band that is available when working with species that have autofluorescent properties.

### *Daphnia* imaging

To better understand the role of vitamin D_3_ as a photoprotectant in invertebrates, confocal fluorescence microscopy was utilized to track the fluorescently labeled D_3_ in *D*. *pulex*. As the *D*. *pulex* have significant body depth, and to determine the uptake and sequestration of D_3_ by the *Daphnia*, it was necessary to perform this experiment *in vivo*. As standard microscope slides would not permit the *D*. *pulex* to move normally, and standard depression well slides allowed too much movement of the *D*. *pulex* for imaging, standard microscope slides were modified for the purpose of this experiment. Plastic spacers of approximately 750 nm– 1 mm in thickness were adhered to slides forming a square the size of a standard cover slip. A cover slip was then adhered to the plastic spacers to create a watertight seal. This “well” allowed enough space to maintain the relative health of the *D*. *pulex* during the imaging, but not enough space for the *D*. *pulex* to continually move out of the focus area. The *D*. *pulex* were imaged using 10x magnification on a Leica TCS SP5 II AOBS Filter-free Tunable Spectral Confocal Research Microscope with Resonant Scanner and Hybrid Detectors (Leica Microsystems Inc., 1700 Leider Lane, Buffalo Grove, IL) attached to a Leica DMI6000 Fully Automated Microscope using Leica LAS system software (RIT, College of Science, Confocal Microscopy Lab).

Optimistically, the fluorescent probe used will have a limited effect on where the biological molecule of interest will be sequestered in *Daphnia*. In other words, unlabeled vitamin D_3_ and the fluorescently labeled vitamin D_3_ (Fig 2: **2**) will be biologically selected for and sequestered equally. In reality, the structural properties of unlabeled and labeled vitamin D_3_ (Fig 2: **2**) are significantly different due to the required bulk of the fluorescent dye compared to the vitamin D_3_ portion of the molecule. To increase our confidence in our results a “control” was run whereby ethylene blue (molecular structure; S2 Fig) was fed to the *Daphnia* and imaged.

*Daphnia magna* were used for the initial imaging experiments due to their size and generally better fitness than *D*. *pulex*. The *D*. *magna* were placed in a 1:10 solution of ethylene blue (without vitamin D_3_; Fig 1) for 30 min and then washed through a series of approximately five synthetic freshwater “baths” to remove excess ethylene blue from their outer carapace. The *D*. *magna* were imaged at 30 min post exposure to determine the fluorescence “noise” that would be associated with imaging of the fluorescently labeled D_3_ in the *D*. *pulex*. The ethylene blue *in vivo* was excited using a 405 nm laser and emission was captured between 600–700 nm using the Leica HyD 12 bit single photon counting detector.

A similar procedure was followed to time-lapse the uptake of the fluorescently labeled D_3_ in the *D*. *pulex*. The *D*. *pulex* were exposed to the labeled D_3_ for 30 min, rinsed through a series of approximately five synthetic freshwater “baths” to remove excess ethylene blue from the outer carapace of the *D*. *pulex*, and imaged for 90 min. This complex of the linked D_3_ in the *D*. *pulex* was excited with a 405 nm laser and emission was captured between 600–700 nm using the Leica HyD 12 bit single photon counting detector (confocal control image; S3 Fig).

## Results

### *Daphnia* fitness

With vitamin D_3_ and UV-A-exposed *D*. *pulex*, survival during the experimental period was not linearly correlated to the concentration of D_3_, but rather D_3_ has a maximum effectiveness on survival at intermediate doses, and may indeed be detrimental in higher doses to the fecundity of the *Daphnia* (survival 62%, 94%, and 53% at 0, 5, and 10 mg D_3_, respectively). The intermediate dose of 5 mg D_3_ in this study showed the highest survival (94 ± 8.7%) of *Daphnia* exposed to chronic UV-A (5 d, 365 nm 115 kJ /m^2^ /nm) and, while 0 mg D_3_ treatments had the highest average reproduction rate in the 5 d experimental period (avg 6 ± 2.8), it is suspected that total reproduction per *Daphnia* over the entire lifespan would be greatest at 5 mg D_3_ (significantly greater survival, 94%, and moderate reproductive rates, 2.7 ± 0.8).

**Fig 3:**
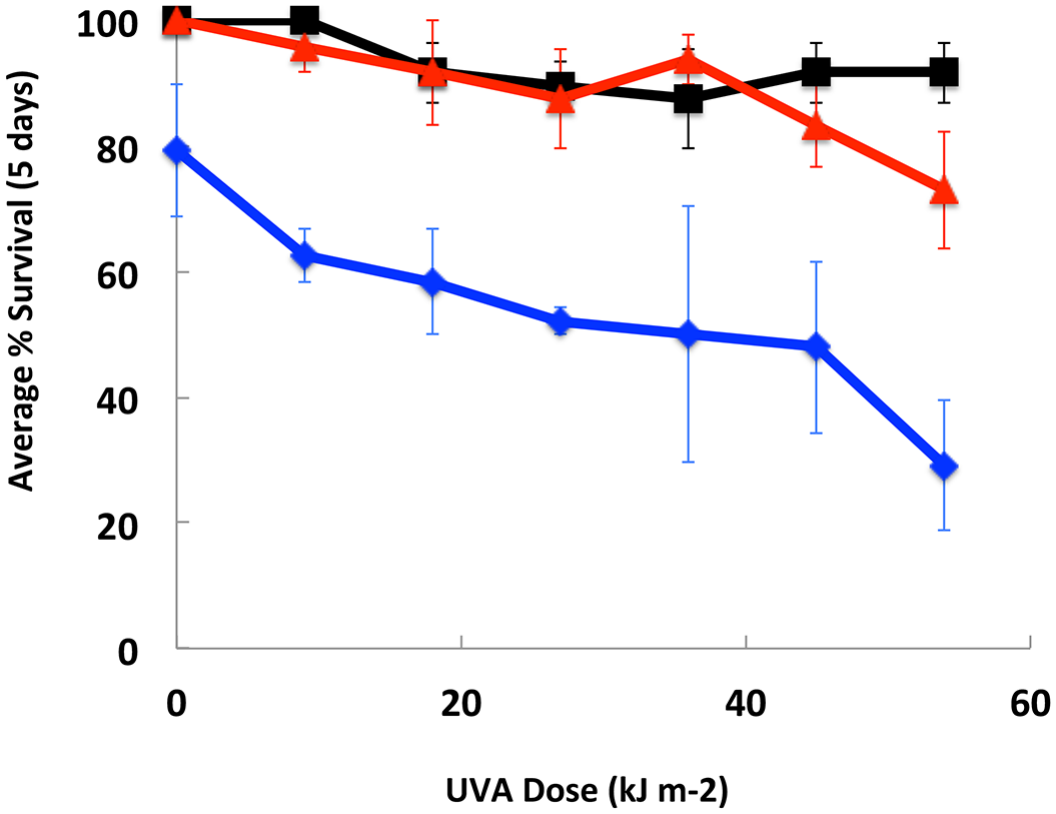
*D*. *pulex* survival with vitamin D_3_. *D*. *pulex* (N = 12) exposed to acute UV-A in the presence of vitamin D_3_. Blue line = 0 mg D_3_; Black line = 5 mg D_3_; Red line = 10 mg D_3_. No reproduction was observed in any individuals during the experimental period. Error = standard error of the mean across 3 trials.

Acute UV-A-exposed *D*. *pulex* trials were conducted to determine survivorship of the *D*. *pulex* under the interaction of UV-A (0–54 kJ/m^2^/nm) and vitamin D_3_ (Fig 3). Again, intermediate concentrations of added vitamin D_3_ (5 mg treatment) showed the maximum effectiveness for nearly all UV-A doses (except 36 kJ/m^2^/nm when 10 mg D_3_ was greatest). This is consistent with our findings in the chronic UV-A exposures (highest average survival at 5 mg D_3_), and the lowest average survival for all doses of acute UV-A being the 0 mg vitamin D_3_ treatment. There was no reproduction in any of the treatments during the 5 d acute UV-A, although low reproduction levels were noted in the 8–10 d old untreated populations of *D*. *pulex* maintained in culture conditions. This is consistent with previous findings of direct effects of acute UV-A on survival mechanisms taxed by the UV-induced stress and repair [27].

In vitamin D_3_ and intermediate acute UV-B (1.59 kJ /m^2^ /nm) *D*. *pulex* trials, survival was not directly correlated to the concentration of D_3_ with percent survival greatest at 10 mg vitamin D_3_ (50 ± 19.1%) and least at 5mg vitamin D_3_ (8 ± 4.3%). Survival was greater in 0 kJ UVB controls in the presence of any vitamin D_3_, 5 mg or 10 mg, than in its absence (100% with 5 and 10 mg vitamin D_3_, versus 83 ± 9.6% with 0 mg vitamin D_3_). There was no survival in the 10 min UV-B treatment (3.18 kJ/m^2^/nm) in this experiment. No reproduction occurred over the 5 d post exposure period, even in individuals with no UV-B exposure. It should be noted that the *D*. *pulex* in this experiment were kept under CoolWhite only lamps (16:8 light:dark cycle) following their UV-B exposure. It has been observed that low levels of light (>25 μEin/m^2^s photosynthetically active radiation (PAR) as quantified using a spherical detector QSL-2101, Biospherical Instruments, San Diego, CA, USA) may be needed physiologically by the *D*. *pulex*. Control studies of this experiment demonstrated adverse effects of low light, and no light, on the fitness of long-term laboratory cultured *Daphnia*. We believe that this result, no reproduction within 5 d following UV-B exposure and slow growth rates even in the no exposure controls, is indicative of this phenomenon based on previous experimentation. This implied decrease in fitness might be explained by changes in the susceptibility of *Daphnia* to parasitic or fungal infections [28].

### Quantification of vitamin D_3_ by HPLC

Percent recovery data are reported as a function of vitamin D_3_ added to the microcosms at the start of each experiment. The modified HPLC technique described herein was optimized to isolate vitamin D_3_ from biological samples. This is a noteworthy advancement from this study as biological samples are notoriously “too dirty” for HPLC, and isolating vitamin D_3_ in such samples is a significant challenge. The chromatograms of the optimized technique are the result of multiple parameter optimizations to isolate D_3_ not only from “dirty” biological samples, but also quantify comparatively miniscule concentrations of the vitamin in many of the microcosm fractions, particularly the *D*. *pulex* (S5 Fig). The ultraviolet absorption spectra peaks at 265 nm and mimics the absorption spectra presented by Hollick [29].

The three fractions isolated from the microcosms described above, *Daphnia*, algae (*Pseudokirchneriella*), and the culture media (“aqueous”), were highly variable in vitamin D_3_ recovery percentage between the fractions themselves (highest recovered percent concentrations of D_3_ remained in the aqueous, 40–42%, versus in the algae, 0.2–1.5%, and *Daphnia*, 0–0.1%). However, the relative concentration of D_3_ in the fractions appears to be independent of quantity of D_3_ added at the initiation of the experiment. The experimental conditions for these trials used the same *Daphnia* and algae setup as described previously, with a chronic UV-A (72 h, 103 kJ /m^2^ /nm) exposure. The percent recovery of D_3_ in the aqueous is consistent under respective conditions (recovery following UV-A = 40.7–42.9%, or without UV-A = 23.7–30.2%), regardless of mg D_3_ added (5mg and 10 mg vitamin D_3_ tested). In the presence of UV-A and 10mg vitamin D_3_, we recovered a greater relative concentration of D_3_ in the algae than under any other experimental conditions (1.5% recovered, versus 0.16% with no UV-A). This may suggest the preferential production and/or uptake of D_3_ by the algae under UV-A-stressed conditions, as compared to those not exposed to UV-A. This supports the hypothesis that vitamin D_3_ may be an important resource under stress conditions. Unfortunately, D_3_ is not detected in sufficient quantities in the *D*. *pulex* in this series to show a direct increase in the uptake of the D_3_, or to associate the quantified uptake with the survival data presented previously.

It is recognized that the quantity of D_3_ in the *Daphnia* may be overestimated in some of our trials described herein, given that the *Daphnia* from the vitamin D_3_ treatments were frozen prior to extraction, not allowing for the metabolic conversion of D_3_ acquired late in the experimental period. However, the relative recovery between fractions is our focus to identify the fundamental pathways for the use of D_3_ in aquatic systems. Ideally, with improved HPLC methodology for the determination of D_3_ oxidation products (e.g. 25-hydroxyvitamin D_3_ 1-α-hydroxylase), we will be able to better address these and other questions in the future.

### Tracking fluorescently tagged vitamin D_3_ in *Daphnia*

**Fig 4:**
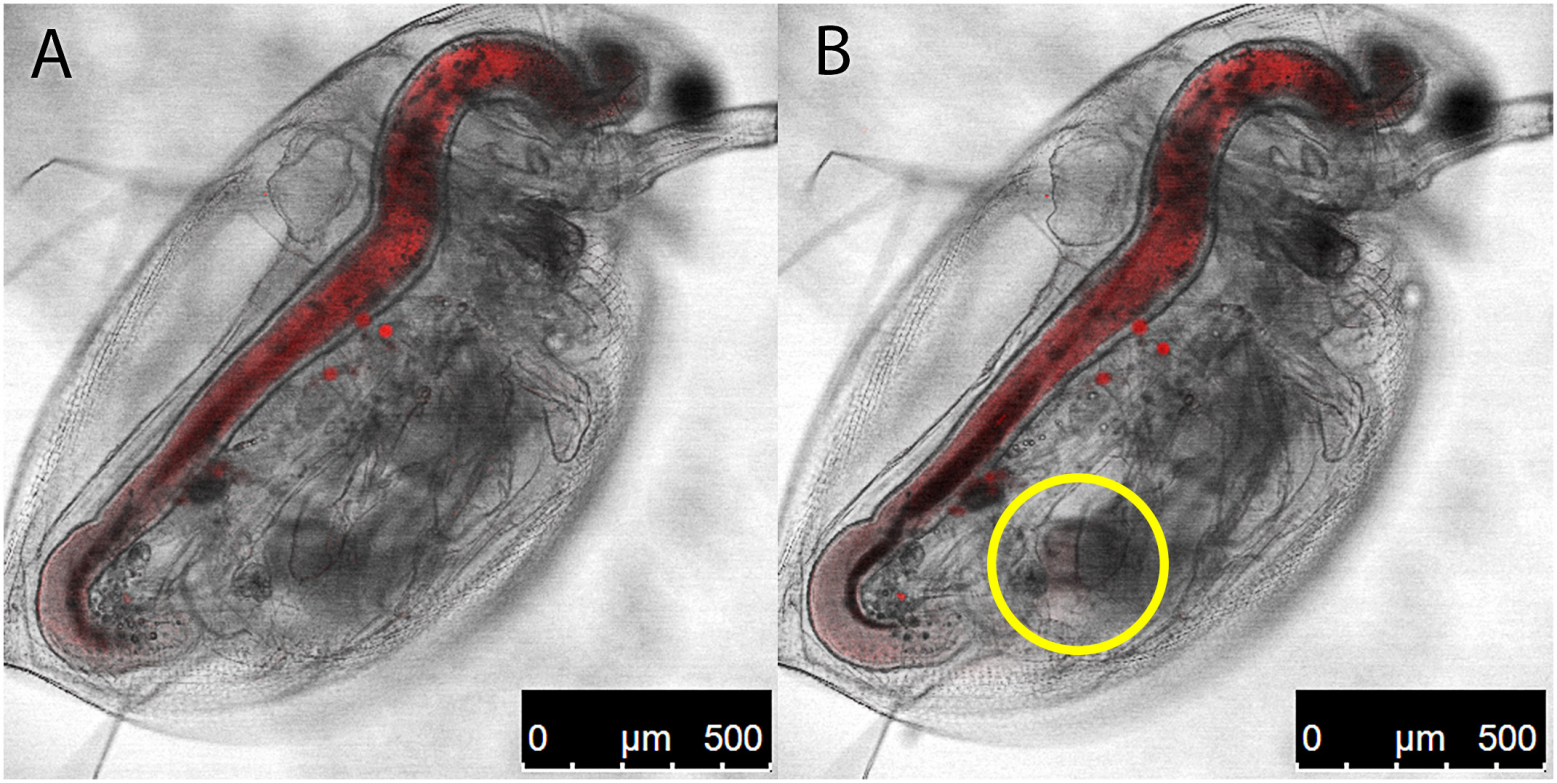
Tracking vitamin D_3_ in *D*. *pulex*. (A) A live *D*. *pulex* was placed in a 1:10 solution of ethylene blue, with no vitamin D_3_ (“control”) and images were captured 30 min post exposure (10x, Leica SP5 Scanning Laser Confocal Microscope). Note the presence of the dye in the gut tract (red stain). (B). Live *D*. *pulex* were placed in a 1:10 solution of ethylene blue linked vitamin D_3_, rinsed thoroughly, and images were captured 120 min post exposure (10x). Image was compiled from a 90-min time lapse to capture sequestration of vitamin D_3_ (red) into the tissues of the *D*. *pulex* from the intestine. The high intensity red “dots” in the image are concentrated dye on the outside of the carapace that was not removed during the rinsing stages. The yellow circle indicates a region where D_3_ sequestration was detected.

All of the fluorescent dye in the control remains in the gut tract, and is ultimately passed out of the *Daphnia*, with no indications of uptake or sequestration of the dye into the surrounding tissues (Fig 4A).

As vitamin D_3_ is transparent by microscopy, the use of a fluorescent dye, such as an analogue of ethylene blue, linked to D_3_ permits observation of the molecule in *D*. *pulex in vivo*. This novel technique is demonstrated through time lapse imaging the sequestration of fluorescently labeled vitamin D_3_ (Fig 2: **2**) in the adductor muscles and carapace of the *D*. *pulex*. As opposed to the control image (Fig 4A), fluorescently labeled vitamin D_3_ is observed moving out of the gut tract and into the surrounding tissues of the *D*. *pulex*. Fig 4B is presented as a direct correlation to the control image (Fig 4A), however, 3D projection still image of the *Daphnia magna* presented herein provides a clearer picture of the linked D_3_ in the tissues surrounding the intestine, including the structural muscles of the carapace. Time-lapse videos of the movement of fluorescently labeled vitamin D_3_ into the carapace and musculature of the *D*. *pulex* and *D*. *magna* are available in the gallery of S.A. Wilbert (http://cias.rit.edu/gallery/photographic-arts-sciences/undergraduate-biomedical-photographic-communications/481).

## Discussion

Any change to the nutrient availability, positive or negative, in a given system will induce a response in the native organisms. With the vast number of nutrients recycled in an ecosystem, and those fluxed in and out of the system over short-term and long-term periods, identifying which are most critical to the survival of any given population can be cumbersome at best. Vitamins can increase the overall fitness of organisms; however, those results vary widely in the literature, with some reported as being detrimental to either longevity of the individuals, or to their fecundity (e.g., [30, 29, 21]). With this multidisciplinary approach to an ecological question, new experiments can be developed to track a single D_3_ molecule from the aqueous environment, through the algae, to the *Daphnia* tissues–the proposed transport pathway in the microcosms described herein. This concept could be extended to consider other vitamins and nutrients critical to the survival of UV-stressed invertebrates in natural systems. Further, through tissue isolation, it would be possible to identify the vitamin D forms most likely to be sequestered and what role they might play in directly increasing a populations’ fitness.

The abiotic stresses under which organisms live on this planet are in constant flux. It is well know that in natural environments freshwater organisms can experience significant diurnal effects of light and temperature, particularly in more temperate regions. Without physiological plasticity and adaptation, organisms under extreme stress environments will demonstrate a significant decrease in fitness over generations, and potentially face extinction. This study demonstrated that vitamin D_3_ increases the overall fitness of *D*. *pulex* exposed to UV-A and UV-B under otherwise controlled conditions (e.g. temperature). Cooler temperatures in shade and at night in freshwater systems will slow the physiological processes of the *Daphnia*. Lower temperatures have been reported to increase the fitness of *Daphnia* following UVR exposure conditions [27] and it is speculated that *Daphnia* fitness would increase significantly if those lower temperatures were combined with the concentrations of D_3_ tested here. An obvious extension of this work would be to not only test the interactive effect of UV, vitamin D_3_ and temperature on *Daphnia* fitness, but to more closely mimic the diurnal fluctuations of surface water temperatures and light conditions in an environmental chamber. This would provide a more comprehensive understanding of the natural uptake, sequestration, and utilization patterns of the vitamin D_3_ by the *Daphnia*, while still maintaining a sufficiently controlled system.

This study has focused on the important role that vitamin D_3_ may play in UVR exposed *D*. *pulex*, but the algae, *Pseudokirchneriella*, must be taken in to account as well. To consider the potentially more important role of algae in a complex ecosystem, an investigation of provitamin complexes and vitamin D_2_ would also be needed. The conversion of provitamins to vitamin D_2_ or D_3_ is highly variable across organisms regardless of trophic level or environment. Ergosterol has been isolated from several green microalgae, diatoms, and chrysophytes [31], and could be converted to bioavailable vitamin D. However, vitamin D_2_ concentrations in another organism, reindeer lichen, are significantly less than D_3_ (when correlated with UVR gradients; [32]). Further vitamin D_2_ is used less efficiently than vitamin D_3_ in some species (e.g. birds; [31]), if it is even used at all. It must be assumed with the prolific occurrence of vitamins D_2_ and D_3_ in the literature that there is a critical role for one or both in most, if not all, organisms. The role is obviously not fully understood in most organisms, potentially due to the lack of clarity surrounding the evolutionary significance of vitamin D between organismal lineages. If vitamin D targets immune system receptors / function, regardless of the type and complexity of the “immune system” present in an organism [31], vitamin D as a mediator of environmental stress should be positively correlated. Future questions should focus on how that mediation is achieved and how it potentially increases the fitness of populations (e.g. bioavailability and specific metabolic pathways).

The next obvious direction for investigation and experimentation would be to consider the bioavailability of vitamin D in ecosystems. Production of vitamin D in the absence of UVR has been reported in waxy-leaf nightshade (*Solanum glaucophyllum*) in the presence of provitamins [33]. This is a fascinating prospect for organisms, such as *Daphnia*, that might be efficiently avoiding significant UVR exposures in the natural systems, and may be dependent on algal sources for vitamin D. But, it also calls into question the following: in the absence of UVR stress, and a concomitant decrease in vitamin D production through photic pathways, why continue to accumulate vitamin D in the organism? What is the immune target specifically in these organisms, and is that target independent / free of UVR damage?

Reports have suggested that mundane chemicals, such as vitamins, can have negative effects in freshwater systems due to their involvement in multiple biological pathways (e.g. lead uptake) [34]. However, we have demonstrated that the presence of vitamin D can play a significantly positive role in abiotic-stressed organisms and are exploring other vitamins and compounds that may play similar roles in such organisms as well. Our HPLC technique is viable and applicable to investigate the variability in aquatic systems, with and without abiotic stress, but requires additional fine-tuning due to the inherent “dirty nature” of biological samples and the low concentrations of target compounds. Further, as we have suggested, the algae may be playing a more significant, direct role in the mitigation of UVR stress in the *D*. *pulex* than we had initially predicted. Future studies should minimally include variable algae species, vitamin D_3_ concentrations, and UVR exposures (natural and artificial) that more closely mimic diurnal conditions.

## Supporting Information

S1 Fig Vitamin D_3_. Molecular structure of vitamin D_3_, the target compound used in this study of *Daphnia* photoprotection from UV radiation.
(PDF)

S2 Fig Ethylene blue. Molecular structure of ethylene blue, the fluorescent compound used as a control in *Daphnia* uptake.
(PDF)

S3 Fig Ethylene blue fluorescence in *Daphnia magna*. *Daphnia magna* was exposed to ethylene blue linked vitamin D_3_ for 30 min, rinsed thoroughly, and fixed in ethanol. Red indicates the presence of the dye-linked vitamin D_3_ in the gut tract and is seen moving into the muscular tissue along the dorsal margin of the anterior end. This still from a 3D Projection, was captured at 10x using a Leica SP5 Scanning Laser Confocal Microscope.
(PDF)

S4 Fig Characterization of vitamin D_3_. Spectroscopic characterization of ethylene blue (solid blue line) and fluorescently labeled vitamin D_3_ (red dotted line).
(PDF)

S5 Fig Detection of vitamin D_3_ by HPLC. High Performance Liquid Chromatography (HPLC) Chromatogram indicating vitamin D_3_ in the standard and in the biological (Aqueous) sample, both at approximately 9.1 min post injection. The standard (top) and aqueous sample (bottom) chromatograms are presented on the left and the right-hand panels show the corresponding optical density ultraviolet absorption spectra. Both ultraviolet absorption spectra peak at 265 nm and mimic the absorption spectra provided by Holick, 2003.
(PDF)
