# Supplementary material for: UV-Stressed Daphnia pulex Increase Fitness through Uptake of Vitamin D3
Source: PLoS One. 2015 Jul 6;10(7):e0131847. doi: 10.1371/journal.pone.0131847 (PMC4492615; doi:10.1371/journal.pone.0131847)
Supplement: S1 Fig — Molecular structure of vitamin D3, the target compound used in this study of Daphnia photoprotection from UV radiation. (PDF) [file pone.0131847.s001.pdf]

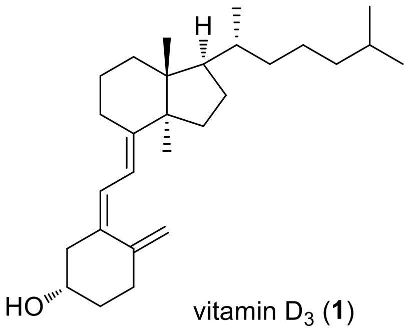

vitamin D_3 (**1**)

S1 Fig. Vitamin D_3. Molecular structure of vitamin D_3, the target compound used in this study of *Daphnia* photoprotection from UV radiation.
