# Supplementary material for: UV-Stressed Daphnia pulex Increase Fitness through Uptake of Vitamin D3
Source: PLoS One. 2015 Jul 6;10(7):e0131847. doi: 10.1371/journal.pone.0131847 (PMC4492615; doi:10.1371/journal.pone.0131847)
Supplement: S2 Fig — Molecular structure of ethylene blue, the fluorescent compound used as a control in Daphnia uptake. (PDF) [file pone.0131847.s002.pdf]

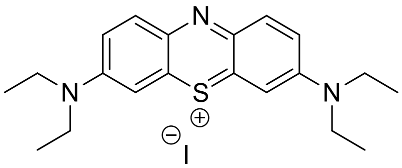
ethylene blue (**5**)

S2 Fig. Ethylene blue. Molecular structure of ethylene blue, the fluorescent compound used as a control in *Daphnia* uptake.
