# Supplementary material for: UV-Stressed Daphnia pulex Increase Fitness through Uptake of Vitamin D3
Source: PLoS One. 2015 Jul 6;10(7):e0131847. doi: 10.1371/journal.pone.0131847 (PMC4492615; doi:10.1371/journal.pone.0131847)
Supplement: S3 Fig — Daphnia magna was exposed to ethylene blue linked vitamin D3 for 30 min, rinsed thoroughly, and fixed in ethanol. Red indicates the presence of the dye-linked vitamin D3 in the gut tract and is seen moving into the muscular tissue along the dorsal margin of the anterior end. This still from a 3D Projection, was captured at 10x using a Leica SP5 Scanning Laser Confocal Microscope. (PDF) [file pone.0131847.s003.pdf]

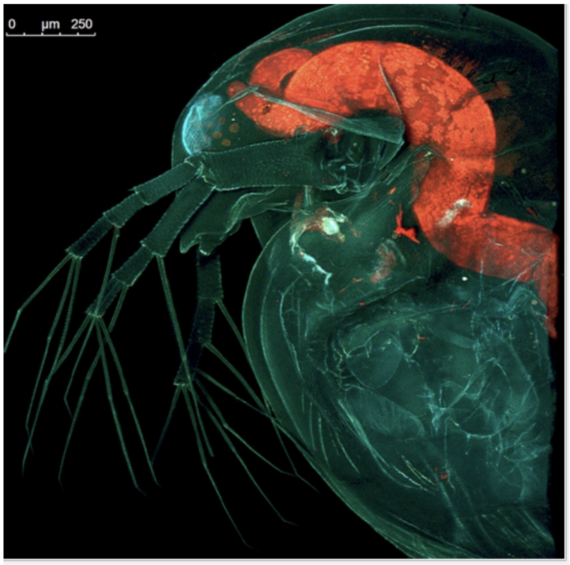

S3 Fig. Ethylene blue fluorescence in *Daphnia magna* . *Daphnia magna* was exposed to ethylene blue linked vitamin D_3 for 30 min, rinsed thoroughly, and fixed in ethanol. Red indicates the presence of the dye-linked vitamin D_3 in the gut tract and is seen moving into the muscular tissue along the dorsal margin of the anterior end. This still from a 3D Projection, was captured at 10x using a Leica SP5 Scanning Laser Confocal Microscope.
