# Supplementary material for: UV-Stressed Daphnia pulex Increase Fitness through Uptake of Vitamin D3
Source: PLoS One. 2015 Jul 6;10(7):e0131847. doi: 10.1371/journal.pone.0131847 (PMC4492615; doi:10.1371/journal.pone.0131847)
Supplement: S4 Fig — Spectroscopic characterization of ethylene blue (solid blue line) and fluorescently labeled vitamin D3 (red dotted line). (PDF) [file pone.0131847.s004.pdf]

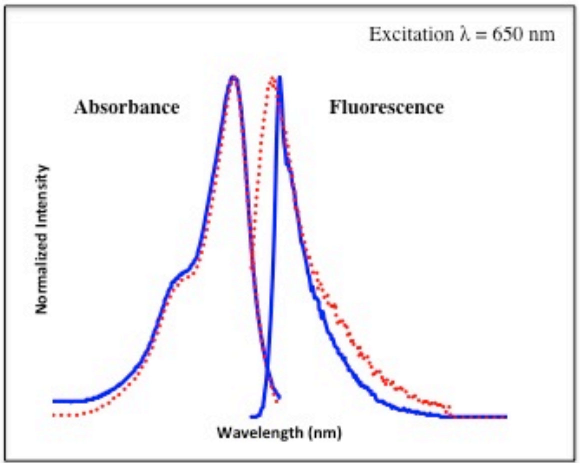

S4 Fig. Characterization of vitamin D_3. Spectroscopic characterization of ethylene blue (solid blue line) and fluorescently labeled vitamin D_3 (red dotted line).
