# Supplementary material for: UV-Stressed Daphnia pulex Increase Fitness through Uptake of Vitamin D3
Source: PLoS One. 2015 Jul 6;10(7):e0131847. doi: 10.1371/journal.pone.0131847 (PMC4492615; doi:10.1371/journal.pone.0131847)
Supplement: S5 Fig — High Performance Liquid Chromatography (HPLC) Chromatogram indicating vitamin D3 in the standard and in the biological (Aqueous) sample, both at approximately 9.1 min post injection. The standard (top) and aqueous sample (bottom) chromatograms are presented on the left and the right-hand panels show the corresponding optical density ultraviolet absorption spectra. Both ultraviolet absorption spectra peak at 265 nm and mimic the absorption spectra provided by Holick, 2003. (PDF) [file pone.0131847.s005.pdf]

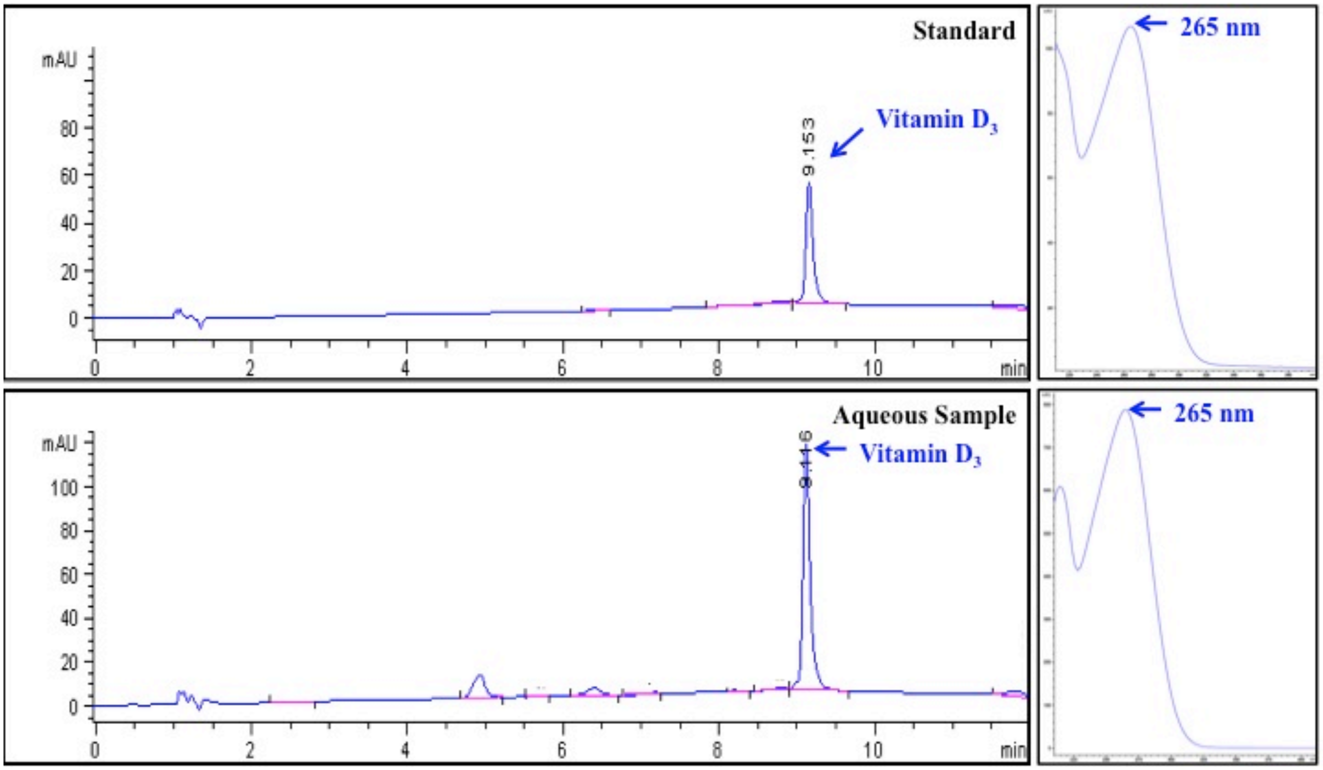

S5 Fig. Detection of vitamin D_3 by HPLC. High Performance Liquid Chromatography (HPLC) Chromatogram indicating vitamin D_3 in the standard and in the biological (Aqueous) sample, both at approximately 9.1 min post injection. The standard (top) and aqueous sample (bottom) chromatograms are presented on the left and the right-hand panels show the corresponding optical density ultraviolet absorption spectra. Both ultraviolet absorption spectra peak at 265 nm and mimic the absorption spectra provided by Holick, 2003.
